# Human MMP28 expression is unresponsive to inflammatory stimuli and does not correlate to the grade of intervertebral disc degeneration

**DOI:** 10.1186/1477-5751-10-9

**Published:** 2011-07-29

**Authors:** Marina Klawitter, Lilian Quero, Alessando Bertolo, Marco Mehr, Jivko Stoyanov, Andreas G Nerlich, Juergen Klasen, Nikolaus Aebli, Norbert Boos, Karin Wuertz

**Affiliations:** 1 Spine Research Group, Competence Center for Applied Biotechnology and Molecular Medicine, University of Zurich, Zurich, Switzerland; 2 Swiss Paraplegic Research, Nottwil, Switzerland; 3 Institute of Pathology, Academic Clinic Munich-Bogenhausen, Munich, Germany; 4 University Hospital Balgrist, Zurich, Switzerland; 5 AOSpine Research Network, Duebendorf, Switzerland; 6 Swiss Paraplegic Center, Nottwil, Switzerland

**Keywords:** MMP28, Epilysin, Matrix metalloproteinase, Intervertebral disc, Inflammation

## Abstract

**Background:**

MMP28 (epilysin) is a recently discovered member of the MMP (matrix metalloproteinase) family that is, amongst others, expressed in osteoarthritic cartilage and intervertebral disc (IVD) tissue. In this study the hypothesis that increased expression of MMP28 correlates with higher grades of degeneration and is stimulated by the presence of proinflammatory molecules was tested. Gene expression levels of MMP28 were investigated in traumatic and degenerative human IVD tissue and correlated to the type of disease and the degree of degeneration (Thompson grade). Quantification of MMP28 gene expression in human IVD tissue or in isolated cells after stimulation with the inflammatory mediators lipopolysaccharide (LPS), interleukin (IL)-1β, tumor necrosis factor (TNF)-α or the histondeacetylase inhibitor trichostatin A was performed by real-time RT PCR.

**Results:**

While MMP28 expression was increased in individual cases with trauma or disc degeneration, there was no significant correlation between the grade of disease and MMP28 expression. Stimulation with LPS, IL-1β, TNF-α or trichostatin A did not alter MMP28 gene expression at any investigated time point or any concentration.

**Conclusions:**

Our results demonstrate that gene expression of MMP28 in the IVD is not regulated by inflammatory mechanisms, is donor-dependent and cannot be positively or negatively linked to the grade of degeneration and only weakly to the occurrence of trauma. New hypotheses and future studies are needed to find the role of MMP28 in the intervertebral disc.

## Background

Proteins of the matrix metalloproteinase (MMP) family play an essential role in tissue homeostasis by initiating breakdown and reorganization of the extracellular matrix. While being tightly regulated in normal physiological processes (e.g. via tissue inhibitors of metalloproteases TIMPs), dysregulation of MMPs has been implicated in many diseases. During intervertebral disc (IVD) degeneration, the expression and activity of a number of MMPs is increased, including MMPs 1, 3, 7, 9 and 13 [1]. Proinflammatory cytokines such as IL-1β and TNF-α as well as bacterial endotoxins (e.g. lipopolysaccharide LPS) can stimulate expression of various MMPs (e.g. MMPs 1, 3, 9 and 13) in the IVD, as well as in cartilage [2-10].

During the recent past, five new members in the MMP family have been identified: MMP24 to MMP28. MMP28, also known as epilysin and most closely related to MMP19, is a soluble MMP that contains an activation sequence recognized by the furin endoprotease following the pro-domain [11]. It is a well-conserved MMP, with great similarity (97%) in the catalytic domain between human and mouse and overall 85% identical amino acids [12]. MMP28 is strongly expressed in testis, as well as in bone, kidneys, lung, heart, colon, intestines, brain, skin and carcinomas [12-17]. It is also expressed in cartilage, synovium and IVDs, with lower expression in bovine discs compared to bovine cartilage [18-22]. Interestingly, MMP28 expression seemed to be increased in osteoarthritis and degenerated IVD compared to healthy tissue, indicating that it may play an important role during these disease processes [18-21].

Despite increasing interest in the role of MMP28 in vivo, little is known about its substrates. Recombinant MMP28 has been reported to degrade casein in vitro and is thought to cleave several neural proteins such as neurite outgrowth inhibitor A ( = Nogo-A), neural cell adhesion molecule ( = NCAM-1) and neuregulin 1 ( = NRG1) [17,23,24]. However, with regard to musculoskeletally relevant proteins, no information on potential substrates is currently available.

As symptomatic degenerated IVDs are characterized by increased levels of certain proinflammatory mediators [10,25-27], which are know to regulate several MMPs [1], we hypothesized that MMP28 expression could be increased in an inflammatory context. Therefore, the aim of this study was to determine the expression level of MMP28 in traumatic or degenerated discs (with different degrees of degeneration) and to investigate the effects of different concentrations of the proinflammatory mediators IL-1β, TNF-α or LPS on its expression in human IVD cells at various time points. Additionally, the effect of the histondeacetylase (HDAC) inhibitor trichostatin A was investigated, as it has been shown to be an up-regulator of MMP28 expression in HeLa cells [28].

## Materials and methods

### MMP28 expression in human IVD biopsies

Thirteen tissue samples from eight patients who had been diagnosed with symptomatic degenerative disc disease or spinal trauma were included in this part of the study. Based on magnetic resonance imaging (MRI) findings, the degree of IVD degeneration was evaluated according to the Thompson grading system prior to the surgical intervention (for detailed information see Table 1) [29]. Informed consent was obtained from all patients according to the local ethical regulations. Frozen biopsies were pulverized, the IVD fragment powder was dissolved in 1 ml of TriFast RNA isolation reagent (PeqLAB) and total RNA was isolated according to the manufacturer's instructions. cDNA was prepared from total RNA using VILO cDNA Synthesis Kit (Invitrogen). For Real-Time (RT)-PCR, cDNA template (5 μl) was mixed with the qPCR reaction solution (IQ SYBR Green Supermix, Bio Rad) and expression of GAPDH and MMP28 was measured:

**Table 1:** Clinical and demographic data of the study population used for ex vivo gene expression analysis (age, sex, diagnosis, Thompson grade [29], vertebral level)

Sample	Sex	Age	Diagnosis	Grade	Level	Region
1	M	31	DDD	II	L5/S1	NP

2a	F	39	Trauma	II	C4/5	AF

2b	F	39	Trauma	II	C4/5	NP

3a	F	57	Trauma	II	Th10/11	AF

3b	F	57	Trauma	II	Th10/11	NP

4a	F	70	Trauma	II	Th12/L1	AF

4b	F	70	Trauma	II	Th12/L1	NP

5a	M	29	Trauma	III	L1/2	AF

5b	M	29	Trauma	III	L1/2	NP

6a	F	34	DDD	III	L4/5	AF

6b	F	34	DDD	III	L4/5	NP

7a	M	41	DDD	III	C4/5	AF

7b	M	41	DDD	III	C4/5	NP

8	M	46	DDD	III	L5/6	NP

9a	M	25	DDD	IV	L5/S1	AF

9b	M	25	DDD	IV	L5/S1	NP

10	M	44	DDD	IV	L5/S1	AF+NP

11	M	50	DDD	IV	L5/S1	AF+NP

12	M	55	Spondylodesis	IV	C3/4	AF+NP

13a	F	58	Spondylodesis	IV	C5/6	AF

13b	F	58	Spondylodesis	IV	C5/6	NP

14	M	37	DDD	V	L5/S1	AF+NP

15a	F	41	DDD	V	L3/4	AF

15b	F	41	DDD	V	L3/4	NP

16a	M	67	DDD	V	L5/S1	AF

16b	M	67	DDD	V	L5/S1	NP

17a	M	72	DDD	V	L4/5	AF

17b	M	72	DDD	V	L4/5	NP

M = male, F = female, NP = nucleus pulposus, AF = annulus fibrosus. DDD = degenerative disc disease, C = cervical, Th = thoracic, L = lumbar, S = sacral (degeneration grade according to Thompson)

GAPDH: Forward-TGGACTCCACGACGTACTCA

GAPDH: Reverse-GGAAGCTTGTCATCAATGGAA

MMP28: Forward-GCCGTGCAGAGCCTGTAT

MMP28: Reverse-GAGTCCCAGGTCTCAAAGTCA

Furthermore, MMP13 was measured as a control gene:

MMP13: Forward-CCAGTCTCCGAGGAGAAACA

MMP13: Reverse-AAAAACAGCTCCGCATCAAC

Primers were used at a concentration of 0.25 nΜ, reactions were carried out in triplicates and the specificity of the amplification products was controlled with a melting curve analysis of each reaction. The 2^-ΔCt ^method was used to calculate gene expression levels of MMP28 and MMP13. To assure consistent PCR quality, a functional cDNA quality control was used. Samples that produced Ct values for GAPDH greater than 26 were not included in the analysis. Instead PCR was repeated with a new sample with identical Thompson grade.

### Isolation, culture and stimulation of IVD cells

Twenty patients who had been diagnosed with symptomatic disc disease or disc herniation (for detailed information see Table 2) and had undergone operative treatment were included in this cell culture study. Informed consent was obtained from all patients according to the local ethical regulations. Disc tissue was minced and treated with 0.3% collagenase NB4 (Serva) and 0.2% dispase II (Roche Diagnostics) in phosphate buffered saline (PBS) for approximately 6 hours at 37°C. After digestion, the cell suspension was filtered using a 70 μm cell strainer (BD Bioscience, Switzerland), centrifuged at 1000 g for 5 min and the cell pellet was washed with and then resuspended in DMEM/F12 (Sigma). Cells were expanded in a 2D culture containing DMEM/F12 (Sigma) with 10% FCS (Tecomedical), penicillin (50 U/mL), streptomycin (50 μg/mL), and ampicillin (125 ng/mL) (Gibco), with medium changes twice a week. When an 80% confluence level was reached, expanded cells in passage 2 or 3 were rendered serum free for 2 hours and, in a first set of experiments, incubated with LPS, IL-1β and TNF-α in a time-dependent (n = 5) and dose-dependent manner (n = 5). For the dose dependency experiment, cells were treated for 18 hours with different concentrations of LPS (0.01 μg/ml, 0.1 μg/ml, 1.0 μg/ml, 2.0 μg/ml), IL-1β (0.1 ng/ml, 1 ng/ml, 5 ng/ml, 10 ng/ml) or TNF-α (0.1 ng/ml, 1 ng/ml, 10 ng/ml, 100 ng/ml). For the time course experiment, cells were incubated with one chosen concentration of LPS (1 μg/ml), IL-1β (5 ng/ml) or TNF-α (100 ng/ml) for 2, 6 or 18 hours in serum-free medium. In a second set of experiments, disc cells as well as HeLa cells (positive control) were incubated with different concentrations of the HDAC inhibitor trichostatin A (1 nM, 10 nM, 100 nM, 1000 nM) (Sigma-Aldrich) for 18 hours (n = 3). As trichostatin A is dissolved in EtOH, a respective EtOH control was included in these experiments. All concentrations of all chemicals were shown to be non-toxic in advance using the MTT assay (data not shown).

**Table 2:** Clinical and demographic data of the study population used for in vitro cell culture experiments (age, sex, diagnosis, Pfirrmann grade [38], vertebral level)

Sample	Age	Sex	Diagnosis	Grade	Level
1	59	F	Disc Herniation	4	L4/5

2	61	M	Disc Herniation	4	L3/4

3	50	M	Disc Herniation	3	L5/S1

4	46	F	Disc Herniation	5	L5/S1

5	51	M	Symptomatic Disc Disease	3	L4/5

6	50	F	Disc Herniation	3	L3/4

7	51	F	Disc Herniation	3	L4/5

8	47	M	Disc Herniation	5	L4/5

9	42	M	Disc Herniation	4	L4/5

10	80	M	Disc Herniation	4	L2/3

11	50	M	Disc Herniation	4	L4/5

12	48	F	Symptomatic Disc Disease	3	L4/5

13	61	F	Disc Herniation	4	L4/5

14	37	F	Disc Herniation	4	L4/5

15	66	M	Disc Herniation	3	L5/S1

16	70	F	Disc Herniation	4	L5/S1

17	40	F	Disc Herniation	4	L4/5

18	55	F	Disc Herniation	3	L4/5

19	26	M	Disc Herniation	4	L5/S1

20	57	M	Disc Herniation	4	L4/5

M = male, F = female, L = lumbar, S = sacral (grading according to Pfirrmann)

### MMP28 mRNA detection in isolated human IVD cells after stimulation

After stimulation, cells were trypsinized and total RNA was isolated according to the manufacturer's recommendation (PureLink RNA Mini Kit, Invitrogen). For each sample, 1 μg of total RNA was reverse transcribed to cDNA (Reverse Transcription Reagents, Applied Biosystems) and then used for real-time RT-PCR measurements using TaqMan Gene Expression assays (Applied Biosystems) for detection of MMP28 (Hs00425233_m1) as well as of TATA-box binding protein TBP (internal control) (Hs00427620_m1). As a positive control, expression of MMP13 was also measured (Hs00233992_m1) on samples stimulated with IL-1β (10 ng/ml), LPS (2.0 μg/ml) or TNF-α (100 ng/ml) for 18 hours.

Gene expression was first normalized to the housekeeping gene before comparing expression of treated cells to untreated control or the respective solvents control if applicable (2^-ΔΔCt ^method). Only changes > 2-fold were considered to be relevant.

### Statistical analysis

To compare gene expression levels between the study groups, the Wilcoxon signed-rank test was used to determine significance between the groups. The statistical software package SPSS was used and the significance level was set to p < 0.05

## Results

### MMP28 gene expression pattern in human disc tissue

**Figure 1:**
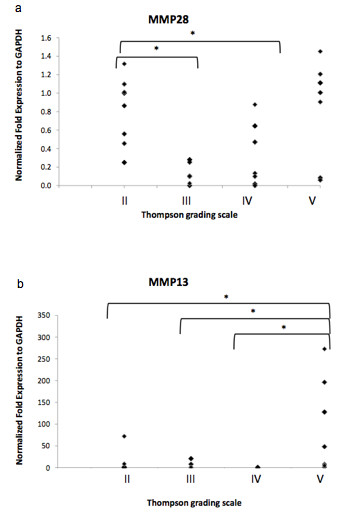
**Ex vivo human IVD tissue gene expression of a) MMP28 and b) MMP13 ( = positive control) in degenerated or traumatic samples with Thompson grading II-V (patients: n = 17, samples: n = 28)**. Data is presented as individual measurement values; * if p < 0.05 between indicated groups.

Analysis of MMP28 gene expression in disc biopsies, which was grouped according to the degree of IVD degeneration (Thompson grade), is shown in Figure 1a: MMP28 was expressed in most of the analyzed disc samples and higher expression levels were found in samples removed because of spine trauma (Thomson grade II = normal adult discs with no disc degeneration). Expression levels were low or practically absent in samples with Thompson grade III (i.e. mild disc degeneration), but increased slightly with increasing disc degeneration, with high donor-donor variation. No consistent statistically significant correlation between MMP28 expression and Thompson grades or disease could be found (Figure 1a). As a control gene, MMP13 expression was analyzed in the same samples, and it showed a strong increase in expression in samples with Thompson grade V degeneration (Figure 1b), as previously described in the literature [30,31].

### Regulation of MMP28 gene expression

**Figure 2:**
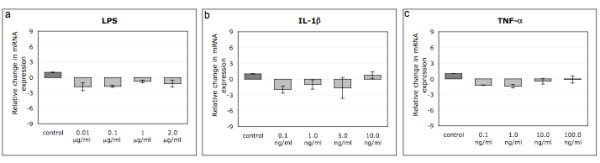
**Regulation of MMP28 gene expression in human IVD cells treated with different concentrations of LPS (a), IL-1β (b) or TNF-α (c) for 18 hours (n = 5 each)**. Data is presented as mean ± SEM; * if p < 0.05 between indicated groups.

**Figure 3:**
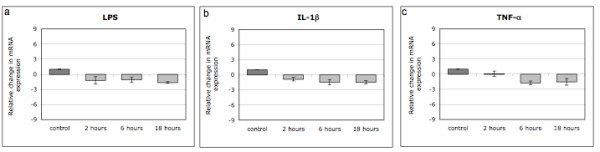
**Regulation of MMP28 gene expression in human IVD cells treated with 1 μg/ml LPS (a), 5 ng/ml IL-1β (b) or 10 ng/ml TNF-α (c) for 2, 6 or 18 hours (n = 5 each)**. Data is presented as mean ± SEM; * if p < 0.05 between indicated groups.

No changes in MMP28 expression could be observed when cells were treated with different concentrations of LPS (Figure 2a), IL-1β (Figure 2b) or TNF-α (Figure 2c) for 18 hours, no matter which concentration was used. As changes in gene expression may strongly depend on the chosen time point, one concentration that is typically used in the literature was chosen for each inflammatory mediator and cellular behavior was investigated after 2, 6 or 18 hours of treatment. However, even at different time points, MMP28 expression was not regulated by LPS (1 μg/ml) (Figure 3a), IL-1β (5 ng/ml) (Figure 3b) or TNF-α (100 ng/ml) (Figure 3c). In order to verify the general responsiveness of disc cells to the chosen treatment conditions, we also measured changes in MMP13 expression. We found that after 18 hour, treatment with IL-1β (100 ng/ml) resulted in a 146.4 ± 28.0 fold increase of MMP13 expression. Similarly, LPS (2.0 μg/ml) caused an 11.1 ± 2.2 fold increase and TNF-α (100 ng/ml) a 134.0 ± 31.5 fold increase in MMP13 mRNA levels (Mean ± SEM, all p < 0.001)(data not shown).

**Figure 4:**
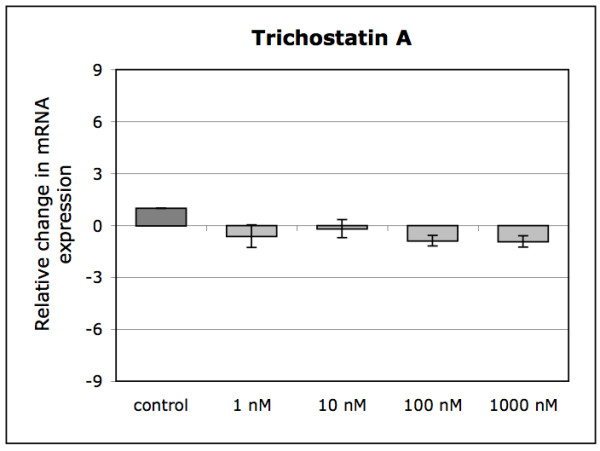
**Regulation of MMP28 gene expression in human IVD cells treated with different concentrations of the HDAC inhibitor trichostatin A for 18 hours (n = 3)**. Data is presented as mean ± SEM; * if p < 0.05 between indicated groups.

Trichostatin A (a HDAC inhibitor) did not cause any changes in MMP28 expression in human IVD cells at any concentration (18 hours only) (Figure 4a). However, in HeLa cells, which were used as a positive control, Trichostatin A caused a significant 2.1 ± 0.1 fold induction of MMP28 expression at 1000 nM (p < 0.001)(data not shown).

## Discussion

Our results indicate that MMP28 is expressed by human intervertebral disc cells in vivo and in vitro, with high donor-donor variations in vivo but did not depend on the level of disc degeneration as measured by Thompson grade score. Additionally, we were able to demonstrate that inflammatory cues (LPS, IL-1β and TNF-α) did not regulate the expression of MMP28 in vitro, indicating that inflammatory processes during IVD disease do not seem to regulate MMP28 expression in vivo.

In our study, MMP28 was expressed in most disc samples with overall more pronounced expression in virtually non-degenerated (grade II), traumatic tissue (removed for the need of spinal fusion after trauma) and severely degenerated IVD tissue. However, for both, non-degenerated tissue and the severe degeneration group, high donor-donor variation was observed. Differences in expression levels in similarly degenerated discs suggest that individual processes during degeneration rather than the degeneration stage itself causes an up-regulation of MMP28. In a study done by Gruber et al., MMP28 was measured on the gene expression level using Affymetrix gene array as well as on the protein level using immunohistochemistry on discs with Thompson grade I to IV [19]. Protein detection of MMP28 expression was also anticipated in our study, but commercially available antibodies proved to be unspecific when performing immunoblotting experiments (data not shown). Comparable to our study, Gruber et al. demonstrated that gene expression of MMP28-precursor (gene identification number AF219624.1) tended to be highest in Thompson grade I and II trauma discs and also elevated in severely degenerated and herniated discs, again without any statistical correlation. Therefore, it is still unclear to date whether and how disc diseases can influence MMP28 expression levels. However, increased levels of MMP28 could be detected in cartilage from osteoarthritis and rheumatoid arthritis patients, suggesting that this novel MMP plays a certain, not completely understood role in some musculoskeletal diseases [18,20,21]. So far, it is not clear why some trauma patients showed high MMP28 expression, but it has been described that certain MMPs such as MMP1 may also increase in disc tissue after traumatic incidences [31,32]. The molecular mechanisms underlying the peculiar expression of MMP28 during trauma and certain cases of more severe degeneration is not clear yet and will have to be analyzed further. During degeneration and trauma, specific molecular events may take place, such as apoptotic or inflammatory processes, changes in matrix protein composition (e.g. increase in collagen type I) and alterations in the mechanical environment [32-36], all of which may explain MMP28 regulation. Aside from MMP28, we also measured MMP13 expression, whose levels have been described in the literature to be elevated with degeneration [30,31]. In our samples, we also found a significant and relatively high increase of MMP13 expression in the grade V degeneration group, compared to all lower grades of degeneration, thus confirming previously published data.

However, when testing whether inflammation regulates MMP28 expression, we could not find any changes in MMP28 mRNA levels after treatment with LPS, IL-1β or TNF-α, although inflammatory mediators regulate many other MMPs (e.g. MMP1, MMP3, MMP9, MMP13), as shown in the literature [7-10]. Indeed, when measuring changes in MMP13 expression in our samples, we were able to detect a significant increase after stimulation with all three agents (LPS, IL-1β or TNF-α). This clearly indicates that the absence of MMP28 regulation observed in this study is not due to lack of sensitivity of our model system. As effects on gene expression after stimulation can depend strongly on the used concentrations as well as on the chosen time point for analysis, variations in dose and sampling points were considered in this study, yet no effects were observed under any condition. In human keratinocytes, TNF-α induced MMP28 at least to a minor degree (up to 8 fold), while multiple other growth factors (bFGF, EGF, GM-CSF, HGF, KGF, PDGF, TGF-β1, VEGF, IGF-1) and cytokines (IFN-γ, IL-1β) did not influence its expression levels at all [37]. All this data indicates that compared to other MMPs, MMP28 seems to be rather unresponsive to external inflammatory stimuli in disc cells, although being expressed in degenerative diseases that are characterized by inflammation. It should however be noted that, in this part of the study, no distinction was made between annulus fibrosus and nucleus pulposus cells as a clear separation of the two zones is not possible in later stage degenerated disc tissue (whereas a separation was possible in less degraded tissue in the first part). Considering the fact that no effect was observed in this mixed cell population, it is however unlikely that a significant alteration would have been observed if distinct cell types had been used.

As TNF-α was not able to induce MMP28 in human IVD cells, we investigated the potential of trichostatin A, a HDAC inhibitor, which was previously shown to strongly regulate MMP28 in HeLa cells. It is assumed that HDAC inhibitors induce MMP28 promoter by acetylation of specificity protein 1 (SP1), which can alter protein-protein interactions and can modify the SP1 containing protein complexes that act at the GC/GT-boxes [28]. However, in our experiments, trichostatin A did not have any effect on the expression levels of MMP28 in disc cells, but the stimulatory effect in HeLa cells could be confirmed in our experimental setting. So far, no other studies have been performed concerning the responsiveness of MMP28 to HDAC inhibitors. Therefore, it is unknown whether most other cell types would show a behavior similar to HeLa cells (regulation) or to IVD cells (no regulation).

## Conclusions

In conclusion, findings of this study provide evidence that MMP28 expression in human IVD tissue is higher in certain cases but the causal relationship between disc diseases and MMP28 expression is unclear to date. In contrast to many other MMPs, MMP28 is not regulated by various inflammatory mediators (IL-1β, TNF-α, LPS) or the HDAC inhibitor trichostatin A. Future studies will be necessary to identify the role of MMP28 in the IVD more conclusively.

## Abbreviations

HDAC: histondeacetylase; IL-1β: interleukin-1β; IVD: intervertebral disc; MMP: matrix metalloproteinase; TNF-α: tumor necrosis factor-α.

## Competing interests

The authors declare that they have no competing interests.

## Authors' contributions

MK and LQ participated in carrying out the cell culture studies, performing the statistical analysis and drafting the manuscript. AB and MM participated in carrying out the analysis of disc biopsies, performing the statistical analysis and corrected the manuscript.

JS participated in carrying out the analysis of disc biopsies, performing the statistical analysis and corrected the manuscript. Additionally, JS participated in the study design and helped coordinating the study. AGN participated in the study design, contributed in data interpretation and corrected the manuscript. JK provided disc biopsies as well as clinical input and corrected the manuscript. NA provided disc biopsies and obtained funding for part of the study. NB participated in the study design, contributed in data interpretation, obtained funding for part of the study and corrected the manuscript. KW is responsible for the study design, coordinated the study, obtained funding and was responsible for writing the manuscript. All authors have read and approved the final manuscript.
